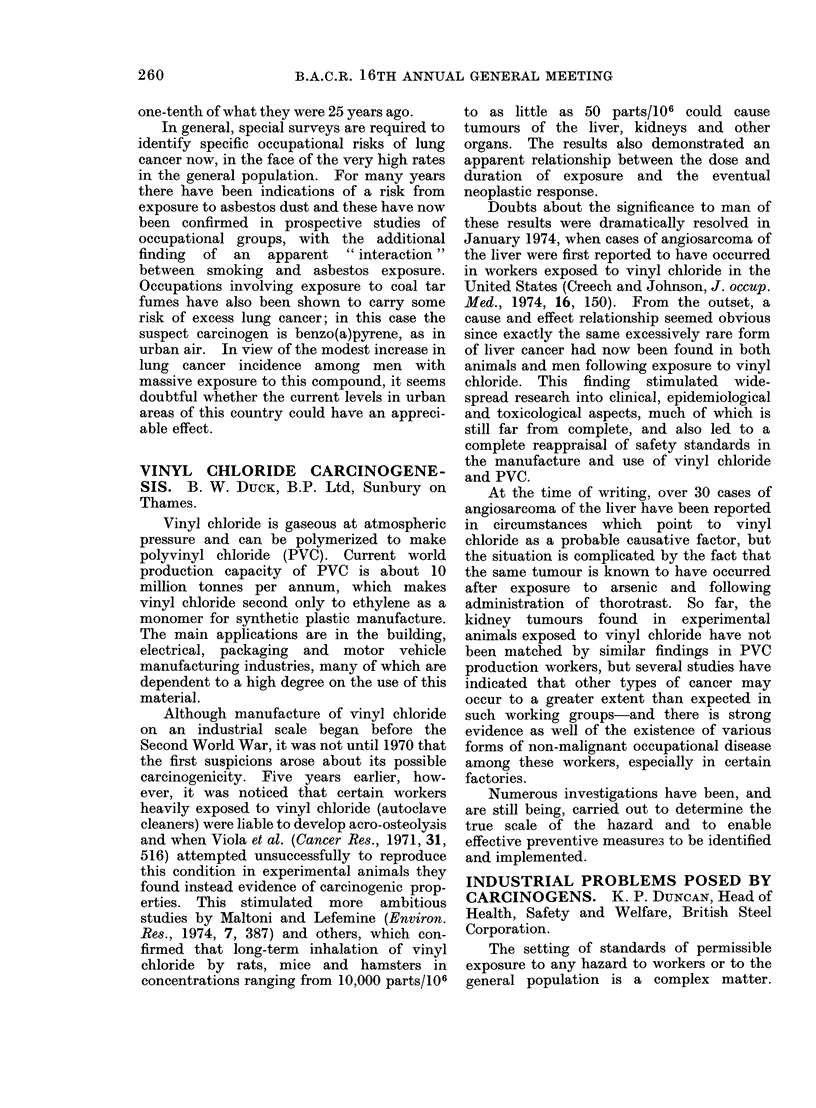# Proceedings: Vinyl chloride carcinogenesis.

**DOI:** 10.1038/bjc.1975.208

**Published:** 1975-08

**Authors:** B. W. Duck


**VINYL CHLORIDE CARCINOGENESIS.** B. W. DUCK, B.P. Ltd, Sunbury on Thames.

Vinyl chloride is gaseous at atmospheric pressure and can be polymerized to make polyvinyl chloride (PVC). Current world production capacity of PVC is about 10 million tonnes per annum, which makes vinyl chloride second only to ethylene as a monomer for synthetic plastic manufacture. The main applications are in the building, electrical, packaging and motor vehicle manufacturing industries, many of which are dependent to a high degree on the use of this material.

Although manufacture of vinyl chloride on an industrial scale began before the Second World War, it was not until 1970 that the first suspicions arose about its possible carcinogenicity. Five years earlier, however, it was noticed that certain workers heavily exposed to vinyl chloride (autoclave cleaners) were liable to develop acro-osteolysis and when Viola *et al.* (*Cancer Res.*, 1971, **31**, 516) attempted unsuccessfully to reproduce this condition in experimental animals they found instead evidence of carcinogenic properties. This stimulated more ambitious studies by Maltoni and Lefemine (*Environ. Res.*, 1974, **7**, 387) and others, which confirmed that long-term inhalation of vinyl chloride by rats, mice and hamsters in concentrations ranging from 10,000 parts/$10^6$ to as little as 50 parts/$10^6$ could cause tumours of the liver, kidneys and other organs. The results also demonstrated an apparent relationship between the dose and duration of exposure and the eventual neoplastic response.

Doubts about the significance to man of these results were dramatically resolved in January 1974, when cases of angiosarcoma of the liver were first reported to have occurred in workers exposed to vinyl chloride in the United States (Creech and Johnson, *J. occup. Med.*, 1974, **16**, 150). From the outset, a cause and effect relationship seemed obvious since exactly the same excessively rare form of liver cancer had now been found in both animals and men following exposure to vinyl chloride. This finding stimulated widespread research into clinical, epidemiological and toxicological aspects, much of which is still far from complete, and also led to a complete reappraisal of safety standards in the manufacture and use of vinyl chloride and PVC.

At the time of writing, over 30 cases of angiosarcoma of the liver have been reported in circumstances which point to vinyl chloride as a probable causative factor, but the situation is complicated by the fact that the same tumour is known to have occurred after exposure to arsenic and following administration of thorotrast. So far, the kidney tumours found in experimental animals exposed to vinyl chloride have not been matched by similar findings in PVC production workers, but several studies have indicated that other types of cancer may occur to a greater extent than expected in such working groups—and there is strong evidence as well of the existence of various forms of non-malignant occupational disease among these workers, especially in certain factories.

Numerous investigations have been, and are still being, carried out to determine the true scale of the hazard and to enable effective preventive measures to be identified and implemented.